# A Genetic Dissection of Natural Variation for Stomatal Abundance Traits in *Arabidopsis*

**DOI:** 10.3389/fpls.2019.01392

**Published:** 2019-11-11

**Authors:** Dolores Delgado, Eduardo Sánchez-Bermejo, Alberto de Marcos, Cristina Martín-Jimenez, Carmen Fenoll, Carlos Alonso-Blanco, Montaña Mena

**Affiliations:** ^1^Facultad de Ciencias Ambientales y Bioquímica, Universidad de Castilla-La Mancha, Toledo, Spain; ^2^Departamento de Genética Molecular de Plantas, Centro Nacional de Biotecnología (CNB), Consejo Superior de Investigaciones Científicas (CSIC), Madrid, Spain

**Keywords:** stomatal abundance, stomatal development, *Arabidopsis thaliana*, natural variation, quantitative trait locus, stomatal lineage, stomatal satellite lineages

## Abstract

Stomatal abundance varies widely across natural populations of *Arabidopsis thaliana*, and presumably affects plant performance because it influences water and CO_2_ exchange with the atmosphere and thence photosynthesis and transpiration. In order to determine the genetic basis of this natural variation, we have analyzed a recombinant inbred line (RIL) population derived from the wild accession Ll-0 and the reference strain Landsberg *erecta* (L*er*), which show low and high stomatal abundance, respectively. Quantitative trait locus (QTL) analyses of stomatal index, stomatal density, and pavement cell density measured in the adaxial cotyledon epidermis, identified five loci. Three of the genomic regions affect all traits and were named *MID* (*Modulator of Cell Index and Density*) 1 to 3. *MID2* is a large-effect QTL overlapping with *ERECTA* (*ER*), the *er-1* allele from L*er* increasing all trait values. Additional analyses of natural and induced loss-of-function *er* mutations in different genetic backgrounds revealed that *ER* dysfunctions have differential and opposite effects on the stomatal index in adaxial and abaxial cotyledon epidermis and confirmed that *ER* is the gene underlying *MID2*. Ll-0 alleles at *MID1* and *MID3* displayed moderate and positive effects on the various traits. Furthermore, detailed developmental studies tracking primary and satellite stomatal lineages show that *MID3*-Ll-0 allele promotes the spacing divisions that initiate satellite lineages, while the *ER* allele limits them. Finally, expression analyses suggest that *ER* and *MID3* modulate satellization through partly different regulatory pathways. Our characterization of *MID3* indicates that genetic modulation of satellization contributes to the variation for stomatal abundance in natural populations, and subsequently that this trait might be involved in plant adaptation.

## Introduction

Gas exchange of terrestrial plants with the atmosphere takes place mostly through stomata, i.e., the microscopic pores that punctuate their otherwise gas-impermeable epidermis (Raven, 2002). Stomatal pores are delimited by two guard cells, whose shape changes in response to physiological and environmental cues dynamically open or close the pore, making stomata behaving as effective valves that regulate gas exchange (Zeiger et al., 1987). The main gases moving through stomata are CO_2_ and H_2_O vapor, which diffuse passively between the leaf internal space and the adjacent atmosphere following concentration gradients. Thus, stomatal opening results in CO_2_ uptake and H_2_O loss or transpiration. This simultaneous gas exchange leads to a physiological conflict or trade-off, since capture of the CO_2_ needed for photosynthesis concurs in space and time with transpiration, making loss of internal water unavoidable (Hetherington and Woodward, 2003). Transpiration, on the other hand, drives water and nutrients uptake by the roots and their xylematic transport, and it refrigerates the plant surface through evaporative cooling (Raven, 2002). As water is often limiting, and the concentration of atmospheric CO_2_ is low, stomata often operate to maximize CO_2_ uptake and moderate water loss, balancing photosynthetic and transpiration rates to prevent desiccation and optimize water use efficiency and growth (Condon et al., 2004; Medrano et al., 2015). Gas exchange balances depend first on stomatal behavior, as guard cell physiology adjusts the degree of stomatal pore opening (Schroeder et al., 2001; Lawson and Blatt, 2014; Murata et al., 2015). However, they also depend on stomata numbers, spatial distribution, and size (reviewed by Bertolino et al., 2019).

Stomatal anatomical features are determined during organ growth, when stomata are gradually formed across the developing epidermis (Geisler and Sack, 2002; de Marcos et al., 2016) under the influence of internal and environmental factors (Casson and Hetherington, 2010; Qi and Torii, 2018). Stomatal development has been deeply studied in the model species *Arabidopsis thaliana* providing precise anatomical and molecular-genetic descriptions (reviewed by Zoulias et al., 2018; Lee and Bergmann, 2019). Three genes encoding related basic helix-loop-helix (bHLH)-type transcription factors drive this process, *SPEECHLESS* (*SPCH*), *MUTE*, and *FAMA* (Ohashi-Ito and Bergmann, 2006; MacAlister et al., 2007; Pillitteri et al., 2007). SPCH initiates the stomatal lineage from a protodermal cell termed meristemoid mother cell (MMC), which experiences an asymmetric division whose smaller product, the meristemoid, undergo repeated asymmetric divisions (MacAlister et al., 2007). The larger products of these asymmetric division are termed stomatal lineage ground cells (SLGCs) and differentiate into pavement cells that surround the stoma to ensure its proper function and occupy most of the mature epidermis (Bergmann and Sack, 2007). Alternatively, SLGCs can experience an asymmetric division termed spacing division and initiate a satellite stomatal lineage that form another stoma away from the primary one (Geisler and Sack, 2002). MUTE directs the late meristemoid to differentiate into a guard mother cell that divides symmetrically (Pillitteri et al., 2007), whereas FAMA drives the differentiation of the twin cell products into guard cells, thus forming the stoma and terminating the lineage (Ohashi-Ito and Bergmann, 2006). These three proteins act together with the bHLHs SCREAM1/ICE1 and SCREAM2 (Kanaoka et al., 2008). In addition, this positively driven process is under the control of complex phosphorylation networks through routes like YODA (YDA)-related MITOGEN ACTIVATED PROTEIN KINASE (MAPK) cascades and brassinosteroids-related BRASSINOSTEROID INSENSITIVE 2 (BIN2) (Bergmann et al., 2004; Gudesblat et al., 2012). Several signaling peptides of the EPIDERMAL PATTERNING FACTORS (EPFs; Torii, 2015) and CLAVATA3/EMBRYO SURROUNDING REGION RELATED (CLE; Qian et al., 2018) families regulate the phosphorylation cascades. These peptides interact, among others, with homo or heterodimers of the membrane receptor TOO MANY MOUTHS (TMM; Nadeau and Sack, 2002) and receptor-kinases of the ERECTA family (ERf; Shpak et al., 2005; Lee et al., 2012). Some of these components are cell-stage specific, short-lived, and act in a combinatorial fashion (Torii, 2012; Lau and Bergmann, 2012), resulting in the orchestrated development of lineages and the formation of precise stomata patterns and abundances.

Direct and specific roles on satellite lineage production has been demonstrated only for a few genes (*AGL16*, *miR824*, *AGB1*, and *GPA1*; Kutter et al., 2007; Zhang et al., 2008). It has been recently established that the SLGC potential for spacing division, impinging on satellite linage formation, is regulated by CLE9/CLE10 and ARR16/17 (Vatén et al., 2018). On the other hand, a number of stomatal development regulators, including TMM, ERf, and EPF1, reinforce correct stomatal patterns regulating stomatal fate acquisition in SLGCs and orienting their asymmetric divisions to place the new meristemoid away from the primary stoma preventing the formation of stomata in contact (Zoulias et al., 2018). However, although *EPF1* and triple *ERf* mutants generate abundant stomata in clusters, their contribution to satellization has not been addressed in detail. Thus, both the number of satellite lineages and the correct stomata patterning during satellization are under the control of these genes. In addition, amplifying divisions contribute indirectly to satellite stomata initiation by regulating the population of SLGCs amenable to undergo spacing divisions and, therefore, regulators involved in amplifying divisions should indirectly impact on satellization.

Previous studies in model and crop plants have established consistent links between stomatal abundance or pattern, and physiological behaviors, which impact water use efficiency and yield in different ways (Bertolino et al., 2019). The latter are relevant target traits for breeding, whose genetic manipulation through determinants of stomatal development is now pursued. Such manipulations include transgenic approaches and induced mutations that modify stomatal numbers and alter physiological performance (reviewed by Bertolino et al., 2019; Endo and Torii, 2019). However, these studies have shown contrasting effects of stomatal density changes, as higher values do not necessarily correlate with increased stomatal conductance and *vice versa* (Dittberner et al., 2018; Bertolino et al., 2019).

In addition to artificial genetic modifications, substantial natural variation has been found for stomata related traits in model and crop plants, such as wheat, soybean, or cotton, which have identified genomic regions conferring advantageous growth through variations of stomatal conductance (reviewed by Faralli et al., 2019). In particular, several studies have taken advantage of the broad natural genetic variation described for anatomical stomatal traits in the model species *A. thaliana* (Woodward et al., 2002; Delgado et al., 2011; Dittberner et al., 2018). This natural variation presumably reflects adaptations to different environmental cues, enabling the identification of wild alleles that have been maintained in natural populations adapted to diverse habitats (Weigel and Nordborg, 2015). Analysis of a core collection of natural accessions revealed considerable genetic diversity not just for anatomical stomatal traits, but also for the developmental pathways underlying stomatal abundance and pattern in mature organs (Delgado et al., 2011). Furthermore, a recent analysis of 330 *Arabidopsis* accessions described significant correlations between anatomical stomatal traits and water use efficiency (Dittberner et al., 2018). Most of this natural variation is quantitative, which indicates that is determined by the simultaneous effect of multiple loci and the environment (reviewed by Alonso-Blanco and Méndez-Vigo, 2014). These loci have been classically addressed by quantitative trait locus (QTL) mapping using mainly recombinant inbred lines (RILs) and introgression lines (ILs) (Wijnen and Keurentjes, 2014; Bazakos et al., 2017; Cockram and Mackay, 2018). However, the recent availability of whole genome sequences for large number of accessions (Cao et al., 2011; The 1001 Genomes Consortium, 2016) has allowed genome-wide association (GWAS) analysis to determine the genetic architecture of complex *Arabidopsis* traits (Atwell et al., 2010; The 1001 Genomes Consortium, 2016; Bazakos et al., 2017; Dittberner et al., 2018). Thus, GWAS analysis of stomatal conductance together with anatomical stomatal traits has shown that natural variation in stomata size is an adaptive trait contributing to the optimization of water use efficiency (Dittberner et al., 2018). Nevertheless, the genetic bases of the natural variation for developmental processes, such as stomatal index or satellization (Delgado et al., 2011), remain unknown.

In this study, we have addressed the genetic bases of the natural variation for stomatal abundance and the underlying developmental processes in *Arabidopsis*. To this end, we have analyzed a RIL population derived from the wild accession Ll-0 and the reference strain L*er*. QTL mapping of cotyledon stomatal index, and stomatal and pavement cell density, identified three loci affecting all traits. To characterize the major effect locus we analyzed in detail multiple natural and induced loss-of-function *er* mutations in different genetic backgrounds, revealing that *ER* dysfunctions have differential and opposite effects on the stomatal traits in adaxial and abaxial epidermis. Moreover, we validated the QTL identified in chromosome 3 [*Modulator of Cell Index and Density (MID) 3*], whose Ll-0 allele leads to a large increase in stomatal numbers. Through genetic and developmental studies, we show that *MID3* and *ER* exhibit additive effects for stomatal abundance traits, and that they have allele-specific effects on the spacing divisions that initiate satellite lineages. Our *MID3* results with indicate that *Arabidopsis* natural variation for stomatal abundance traits is partly determined by genetic modification of satellization, a highly specific event during stomatal lineage development.

## Material and Methods

### Plant Material and Growth Conditions

*A. thaliana* accessions and mutants were obtained from the *Arabidopsis* Biological Resource Center (ABRC), the Nottingham *Arabidopsis* Stock Center (NASC), or the National Institute of Versailles's Agronomic Research (INRA, France). The Landsberg strain carrying wild-type *ERECTA* (*ER*) allele (here designated as L*ER*) was provided by Dr. M. Koornneef (Wageningen University, the Netherlands). The population of 139 RILs derived from a cross between Landsberg *erecta* (which carries *er-1* mutation) and the Llagostera-0 wild accession (L*er* x Ll-0 RILs) has been previously described (Sánchez-Bermejo et al., 2012). The loss-of-function mutant alleles used for *ER* were described previously: *er-1* (Rédei, 1962; Torii et al., 1996), *er-105* (Torii et al., 1996) and *er-123* (Lease et al., 2001).

For the genetic validation of *MID3*, two near isogenic lines (NILs) carrying *MID3*-Ll-0 region were developed in L*ER* (NIL1) or L*er* (NIL2) backgrounds. These NILs were derived by crossing LLL90, which carries 30 and 70% Ll-0 and L*er *genome proportions, with L*ER*. The F_2_ (LLL90xL*ER*) progeny was genotyped to select a plant heterozygous for the *ER* locus (*ER*/*er*-1), homozygous for *MID3*-Ll-0 region and with no other Ll-0 introgression. NIL1 and NIL2 were selected from the self-progeny of this plant, as homozygous for a single introgression around the *MID3*-Ll-0 region (between positions 1.8 and 7.9 Mb of chromosome 3) in the L*ER* and L*er* backgrounds, respectively. For *MID3* fine-mapping, NIL1 was backcrossed with L*ER* and the derived F_2_ was used to develop six ILs referred to as STAIR (ST) lines, which were homozygous for partially overlapping Ll-0 fragments of the *MID3* region. Thereafter, the ST6 line, bearing a Ll-0 fragment between positions 6.35 and 7.5 Mb, was backcrossed again with L*ER* to develop four additional homozygous ILs carrying smaller LL-0 introgression fragments and referred to as mini-ST lines.

Plants were grown in a greenhouse supplemented with lamps to provide a long‐day (LD) photoperiod (16 h light/8 h dark) at 18–23°C, as previously described (Sanchez-Bermejo et al., 2012) or in growth-chambers (Conviron MTR30) set-up at 21 ± 1°C, 60% relative humidity, and 150 ± 20 µmol m^−2^s^−1^ irradiance (Delgado et al., 2011). Seeds were sown in Petri dishes containing a filter paper soaked in water and stratified 4 days at 4°C in darkness. Thereafter*,* Petri dishes were transferred to a growth chamber for four additional days to allow germination, and seedlings were then planted on pots containing soil:vermiculite in proportions 3:1. Pots were moved to greenhouse or growth chambers depending on the experiments.

For the epidermal phenotyping of the RIL population, all RILs and parents were grown simultaneously in the greenhouse, in a single experiment. The *MID3* NILs, ST, mini-ST lines, and related F_1_ plants were grown along their reference genotypes in several greenhouse and/or growth chamber experiments. All phenotyping experiments were organized in three complete randomized block designs, with one pot per line and block, and five to six plants per pot.

### Quantitative Analysis of Epidermal Phenotypes and Stomatal Lineages

Stomatal and pavement cell densities (SD and PD) and stomatal index (SI) were scored in mature cotyledons, using the dental resin method as previously described (Delgado et al., 2011). Epidermal cell counts of each individual plant were an average from two 0·327-mm^2^ areas at the median region of the cotyledon. For evaluation of the RILs, three individuals per genotype were scored. In the rest of experiments, 10–20 plants of each genotype were used. SD and PD were calculated as number of stomata or pavement cells per area unit (cell number mm^−2^), respectively, and SI as percentage of epidermal cells that were stomata (number of stomata/total number of epidermal cells × 100).

Primary and satellite stomatal lineages were scored as in Delgado et al. (2011). In brief, cotyledons were collected at 3 or 5 dag and fixed in ethanol:acetic acid 9:1 (v/v), dehydrated through ethanol:water series, rehydrated, and mounted in chloral hydrate:glycerol:water (8:1:2, w/v/v) clearing solution. The adaxial epidermis was inspected with differential interference contrast (DIC) under a Nikon Eclipse 90i upright microscope and a DXM1200C camera for image acquisition. The different cell types were identified and scored. Lineage initiation was monitored by the primary lineage index (PLI) or proportion of primary stomata plus primary stoma precursors to total epidermal cells, and the satellite lineage index (SLI), or proportion of satellite stomata plus satellite stomata precursor. From this values, the total lineage index (TLI = PLI + SLI) and the percentage of satellite lineages (%SL = SLI/TLI) were calculated. Reiteration of satellite lineages was monitored as the proportion of primary lineages producing a satellite lineage (%PLS) and the proportion of satellite lineages reiterating satellization (%SLS). In addition, SI was determined in mature cotyledons of 10 plants simultaneously grown in same chambers.

Environmental interaction of *MID3* effects on SI, SD, and PD were evaluated by a two-factor analysis of variance (ANOVA), with genotype (NIL1) and environment (green house *vs.* growth chamber) as fixed factors. Differences between mean trait values or stomatal lineage indices were tested by Student's *t*-tests. All statistical analyses were performed with the SPSS v. 24 package (SPSS Inc., Chicago, IL, USA).

### Genotyping and Gene Sequencing

DNA for genotyping was prepared according to Edwards et al. (1991). Plants were genotyped with markers previously reported (Sánchez-Bermejo et al., 2012) or newly developed within *MID3*-region, or at specific alleles of *ER* (*er-1*, er-Van-0 and *ER*), *FAMA, MUTE*, and mitogen-activated protein kinase kinase 5 (*MKK5*). For new markers, public resources (Nordborg et al., 2005; Cao et al., 2011) were used to design INDELS, CAPS, or dCAPS markers (**Table S1**).

The coding region of *MKK5* (1,046 bp) was sequenced in Ll-0, L*er*, and L*ER* using DNA extracted with the DNeasy Plant Mini Kit (Qiagen). The *MKK5* region was amplified by polymerase chain reaction (PCR) with the HiFi PCR Kit (KapaBiosystems) using specific primers (**Table S2**). The PCR products were purified with the Illustra GFX PCR Purification Kit (GE Healthcare) and sequenced with BigDye technology.

### QTL Anaylis

QTL mapping was carried out separately for each trait using mean RIL values that were previously log_e_ transformed for cell density traits (SP and PD) or arcsin-root transformed for the SI. QTL were located by the multiple‐QTL‐model method (MQM) implemented in MapQTL v. 4.0 software (Van Ooijen, 2000). A logarithm of the odds (LOD) thresholds of 2.4 was used for QTL detection, corresponding to a genome‐wide significance *α* = 0.05 as estimated with MapQTL permutation test. The additive allele effect and the percentage of variance explained by each QTL, as well as the total variance explained by the additive effects of all QTL detected for each trait, were obtained from MQMs. Additive allele effects correspond to half the differences between the estimated means of the two RIL genotypic groups.

Two‐way genetic interactions were tested by two‐factor using the markers linked to detected QTL. The percentage of variance explained by significant interactions was estimated by type III variance components analysis. The total variances explained for each trait, including additive and interaction effects, were estimated from general linear models including all significant effects from the detected QTL. Broad sense heritabilities (*h* *^2^* *_b_*) were estimated as the variance component among RILs derived from type III ANOVAS. Statistical tests were performed with SPSS v. 24 package (SPSS Inc., Chicago, IL, USA).

### RNA Extraction and qPCR Analysis

Cotyledons were collected at 3 dag and RNA was extracted from three independent biological replicates with TRIzol (Invitrogen), followed by column purification with the High Pure RNA Extraction Kit (Roche Diagnostics). cDNA was synthesized with the High-Capacity cDNA Reverse Transcription Kit (Applied Biosystems) according to the manufacturer's instructions. qPCR reactions were performed with the Maxima SYBR Green qPCR Master Mix (Thermo Scientific), and run in a LightCycler 480 Instrument (Roche Diagnostics). Relative expression changes were determined using the LightCycler^®^ 480 Software Version 1.5 (Roche Diagnostics). *ACT2* (At3g18780) and *UBQ10* (At4g05320) were used as reference genes. The primer sets used are given in the **Table S2**.

## Results

### Genetic Variation for Stomatal Abundance Traits in L*er*, Ll-0, F_1_ Hybrids, and the L*er *x LI-0 RIL Population

**Figure 1:**
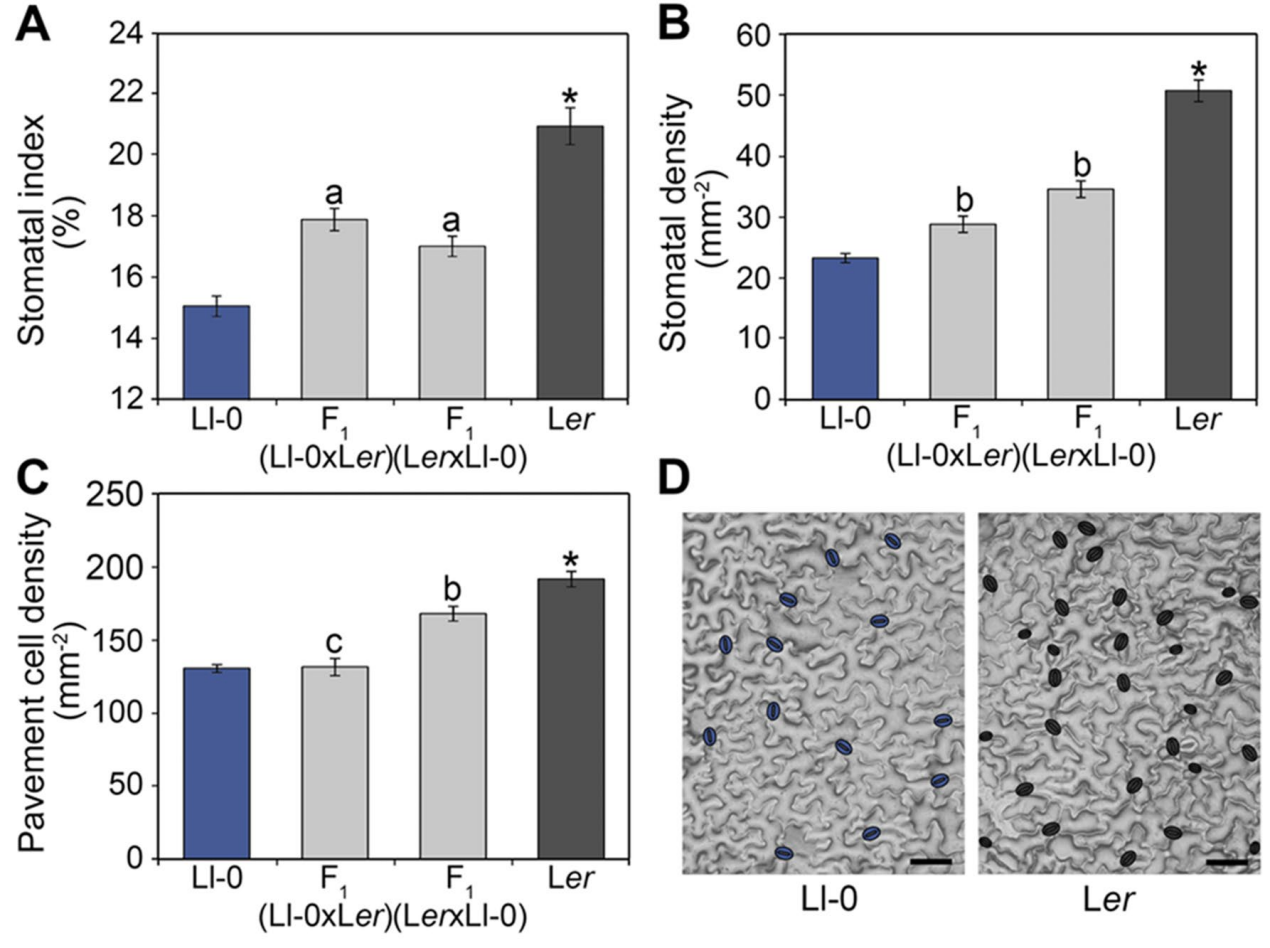
Stomatal abundance traits in Ll-0, L*er*, and their reciprocal hybrids. Adaxial cotyledon epidermis was scored at maturity (21 dag) for stomatal index **(A)** and density **(B)** and for pavement cell density **(C)**. Bars represent mean ± SE of 10 plants. Phenotypic differences were analyzed using a Tukey's test. Significant differences between Ll-0 and L*er* are indicated with an asterisk. Letters over bars refer to phenotypic comparisons of each F_1_ hybrid with the remaining genotypes: "a" indicates significant differences from both parents; "b" indicates differences from all other genotypes; "c" indicates differences from male parent and reciprocal hybrid. **(D)** Representative mature adaxial cotyledon epidermis of Ll-0 and L*er*, with stomata false-colored in blue. Micrographs were obtained with the dental resin method. Scale bars are 100 µm.

The large quantitative variation previously described for stomatal numbers among natural *A. thaliana* accessions (Delgado et al., 2011) indicates that stomatal abundance is under multigenic control. To investigate the genetic bases underlying this variation we selected the Llagostera-0 accession (Ll-0) and the reference strain Landsberg *erecta* (L*er*) since preliminary analyses identified substantial phenotypic differences and they are the parents of an existing RIL population (Sánchez-Bermejo et al, 2012). Phenotypic analyses of both lines were assessed in the adaxial epidermis of fully expanded cotyledons for three stomatal abundance related traits, namely stomatal index (SI), stomatal density (SD), and pavement cell density (PC). Values of the three stomatal abundance traits were strikingly lower in Ll-0 than in L*er* (**Figure 1**), with a range of variation similar to that observed among extreme phenotypes in representative samples of other natural accessions (Delgado et al., 2011).

To determine the overall mode of inheritance and the dominance of stomatal phenotypes we also measured the abundances of the different epidermal cell-types in F_1_ hybrids derived from reciprocal crosses between Ll-0 and L*er* (**Figure 1**). The F_1_ hybrids obtained using Ll-0 or L*er* as mother plant showed similar SI, with intermediate values between both parental lines. Thus, the low Ll-0 SI appeared determined by the zygotic genotype and acts semidominantly. By contrast, the reciprocal F_1_ hybrids differed significantly for SD and PD, indicating an effect of the maternal genotype on these traits. Nevertheless, F_1_ hybrids displayed an intermediate phenotype between the two parental lines for SD, but a similar phenotype to mother plants for PD. Therefore, PD seems determined mainly by the maternal genotype whereas SD is under the control of both the zygote and mother genotypes.

**Figure 2:**
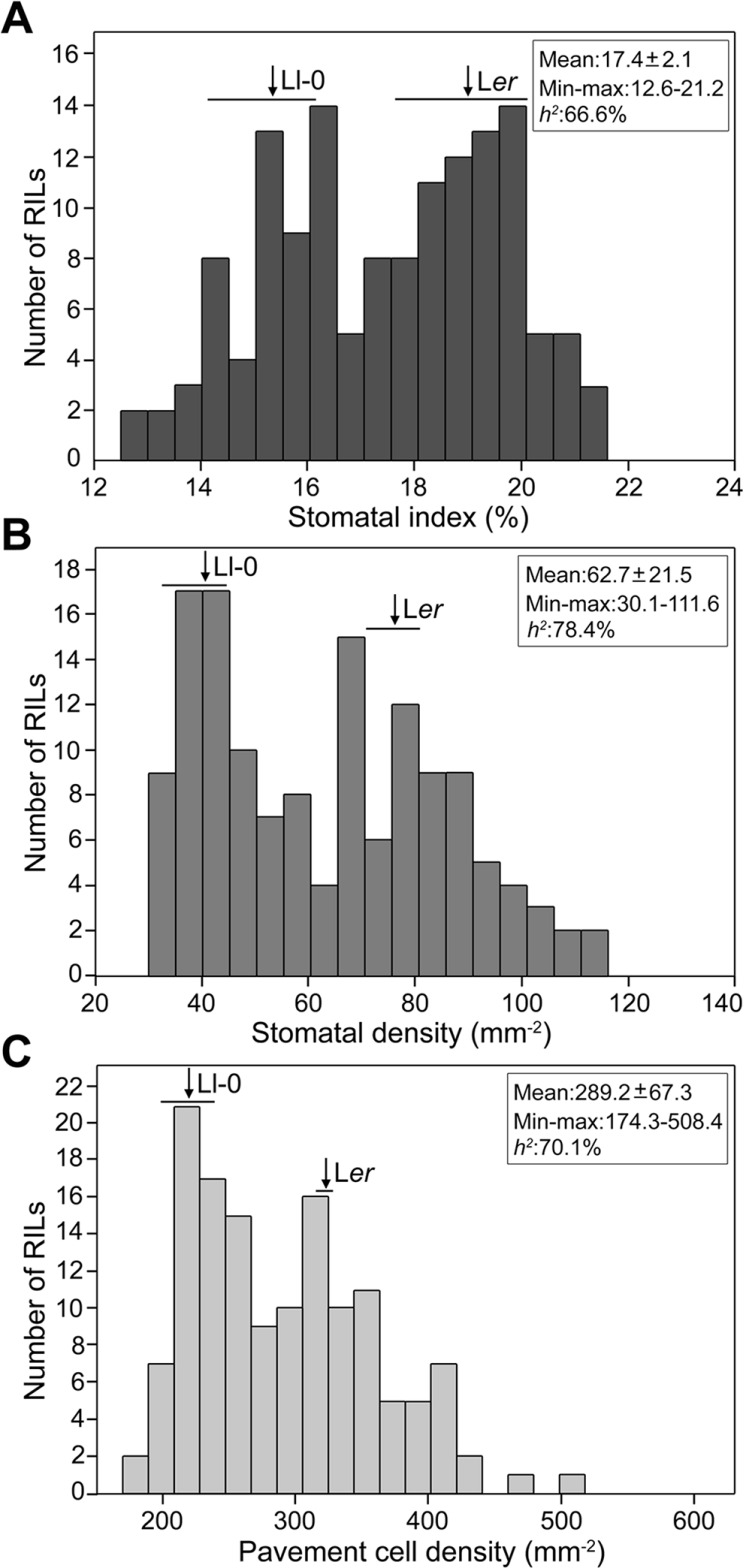
Frequency distributions of stomatal abundance traits in the L*er x* Ll-0 (RIL)population. Stomatal index **(A)** and density **(B)** and pavement cell density **(C)** were scored in mature adaxial cotyledon epidermis of green-house grown plants. Arrows and horizontal bars mark mean ± SD of parental lines. The population mean, the minimum and maximum RIL means, and the broad sense heritability (*h* *^2^*) of traits are indicated inside each panel.

To establish the genetic bases of the differences between Ll-0 and L*er* in epidermal cell-type abundance we measured SI, SD, and PD in adaxial cotyledon epidermis in a L*er* x Ll-0 RIL population of 139 lines (**Table S3**). Broad sense heritabilities varied between 78.4% for SD and 66.6% for SI, with substantial transgressive segregation beyond both parental values appearing for all traits (**Figure 2**). In addition, the three traits were highly and positively correlated within the RILs (**Figure S1**), as observed for natural accessions (Delgado et al., 2011). Cell-density traits (SD and PD) showed a stronger correlation (*r* = 0.96; *P* < 10^−77^) than stomatal abundance traits (SI and SD; *r* = 0.88; *P* < 10^−45^), while SI and PD had the lowest correlation strength (*r* = 0.72; *P* < 10^−23^). These results suggest that the three stomatal traits share a large portion of their genetic bases, which involve alleles increasing and decreasing them in both parental accessions.

### Quantitative Trait Locus Analysis in the L*er *x Ll-0 RIL Population

**Figure 3:**
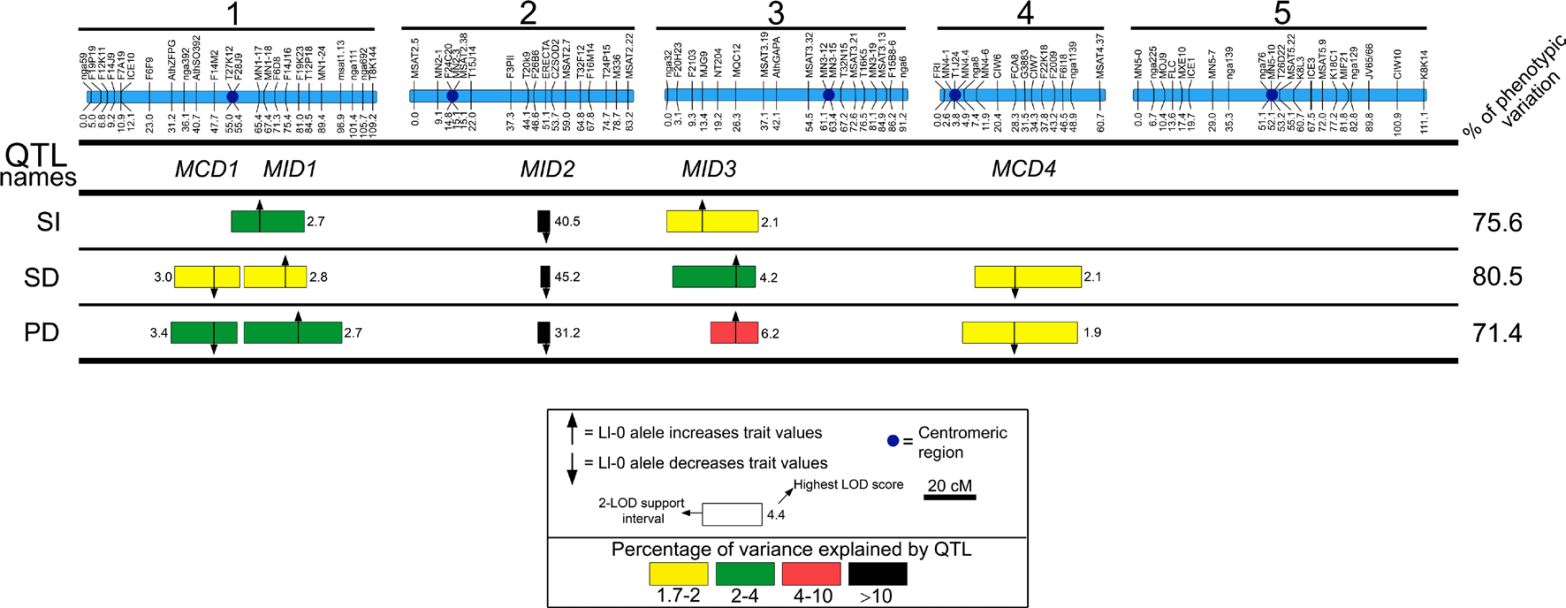
QTL mapping of stomatal abundance traits in the L*er*/Ll-0 RIL population. Blue bars on the top represent the genetic maps of the five chromosomes, whereas QTL names are shown below: *MID, Modulator of Cell Index and Density; MDC*, *Modulator of Cell Density*. Horizontal lines separate traits analyzed: stomatal index (SI), stomatal density (SD), and pavement cell density (PD). Numbers in the right side show the percentage of phenotypic variance explained by the additive effects of all detected QTL. For each trait, the locations of QTL identified are shown as 2-LOD support intervals. Position of arrows and numbers inside boxes correspond to the highest LOD scores. Colors of QTL boxes depict the different ranges of QTL-explained variances as described in the inset. Arrows indicate that the additive effect of Ll-0 alleles increase (pointing up) or decrease (pointing down) the trait values in comparison with L*er* alleles.

To identify the loci that contribute to the described phenotypes, we performed QTL analyses for each trait. In total, five genomic regions affecting two or three of the traits were detected as accounting for 75.6%, 80.5%, and 71.4% of the variation for SI, SD, and PD, respectively (**Figure 3** and **Table S4**). Three genomic regions located on chromosomes 1, 2, and 3 affected all traits suggesting pleiotropic effects of these three loci on cell-type proportion and density. Thus we named these regions *MID* (*Modulator of Cell Index and Density*) 1 to 3 according to their chromosomal location. The other two genomic regions, located on chromosomes 1 and 4, affected both SD and PD suggesting the presence of loci specifically regulating cell size processes, and were named as *MCD* (*Modulator of Cell Density*) 1 and 4.

*MID2* showed very large effects explaining between 51.7 and 67.6% of the phenotypic variation for all traits, with the Ll-0 allele reducing cell-type abundance values. The remaining regions had small relative effects (<5%); Ll-0 alleles at *MID1* and *MID3* displayed positive effects on the various traits, whereas at *MCD1* and *MCD4* Ll-0 alleles decreased cell densities. Hence, both parental genotypes carry alleles increasing and reducing each trait, in agreement with the transgressive segregation observed in the RIL population. Moreover, the co-location of QTL affecting the three traits in the various *MID* genomic regions explains the strong correlation among cell proportion and cell density traits in this population.

Finally, no significant two-way genetic interaction (*P* > 0.01) was found among these regions for any trait, supporting that differences in stomata and pavement cell abundance among L*er* and Ll-0 are mainly determined by additive effects of a small number of loci.

### Phenotypic Characterization and Candidate Gene for *MID2*

**Figure 4:**
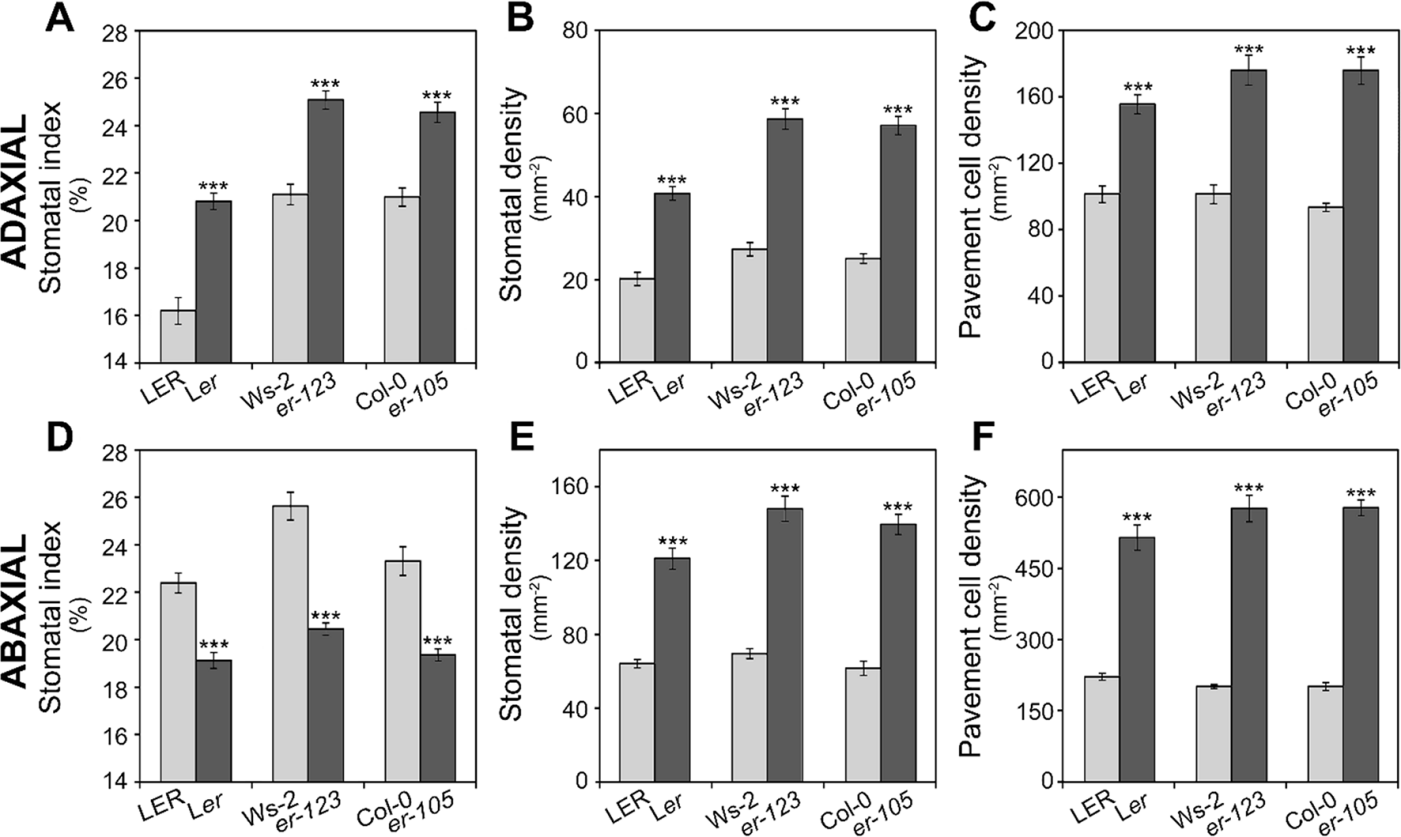
Effect of *erecta* alleles on stomatal abundance traits in both cotyledon epidermis. Stomatal index and density and pavement cell density were scored in adaxial **(A**–**C)** or abaxial **(D**–**F)** cotyledon epidermis of indicated genotypes. L*ER*, Col-0, and Ws-2 carry wild-type *ERECTA* alleles, whereas L*er*, *er-105*, and *er-123* harbor dysfunctional *erecta* alleles. For each trait, the mean ± SE of 10–20 plants are presented. Asterisks indicate significant differences (*P* ≤ 0.001; Student's *t*-test) between a given *er* mutant and the corresponding wild type.

*MID2* mapped centered on *ER*, which segregates in the L*er* x Ll-0 mapping population because L*er* parental carries the *er-1* mutant allele while Ll-0 harbors an *ER* functional allele. Since it has been shown that *erecta* mutations in Col and Landsberg backgrounds strongly affect stomatal abundance traits in the abaxial epidermis of cotyledons and adult leaves (Masle et al., 2005; Shpak et al., 2005; Tisné et al., 2008) *ER* might underlie *MID2*. To test this hypothesis we evaluated the effects of *er-1, er-105*, *and er-123* loss-of-function mutations in L*er*, Col, and Ws-2 genetic backgrounds, respectively, on stomatal abundance traits in adaxial and abaxial cotyledon epidermis (**Figure 4**). The three *er* mutations largely increased adaxial SI, SD, and PD compared to their corresponding wild-type alleles (**Figures 4A–C**), thus indicating that *ER* underlies *MID2*. In agreement with earlier studies (Shpak et al., 2005), we also found that these *er* mutations lowered SI whereas they increased SD and PD in the abaxial epidermis of cotyledons (**Figures 4D–F**). The decrease in abaxial SI is partly due to the abundant small cell patches that result from arrested lineages, which did not produce stomata (see *Discussion*). **Figure S2** shows representative adaxial and abaxial epidermis of *er-105* cotyledons where the arrested lineages are patent in the abaxial side but absent in the adaxial epidermis. Therefore, *ER* dysfunction has differential and opposite effects on cell-type proportions in adaxial and abaxial cotyledon epidermis.

We further tested if the natural *erecta* null allele in the Vancouver-0 (Van-0) accession (van Zanten et al., 2009) is also involved in determining the very high SI, SD, and PD on adaxial cotyledon we previously described for this genotype (Delgado et al., 2011). An allelism test between Van-0 and *er-1* was performed using L*er* and L*ER* as sources of mutant and wild-type *ER* alleles (**Figure S3**). Van-0 displayed significantly higher SI, SD, and PD than L*er* (*P* ≤ 0.01 for all traits; Student's *t*-test). Reciprocal F_1_ hybrids derived from crosses between Van-0 and L*er* showed similar low values to L*er* for cell density traits, but high and similar to Van-0 for SI. By contrast, the reciprocal Van-0/L*ER* F_1_ hybrids displayed low SD and PD values as L*ER*, thought they had a SI phenotype intermediate between both parents (**Figure S3**). These results indicate an overall dominance of the *ER* functional allele for cell density traits but semidominance for cell-type proportion, although additional loci likely contribute to the L*ER*/L*er*/Van-0 variation.

### Genetic Validation of *MID3*

**Figure 5:**
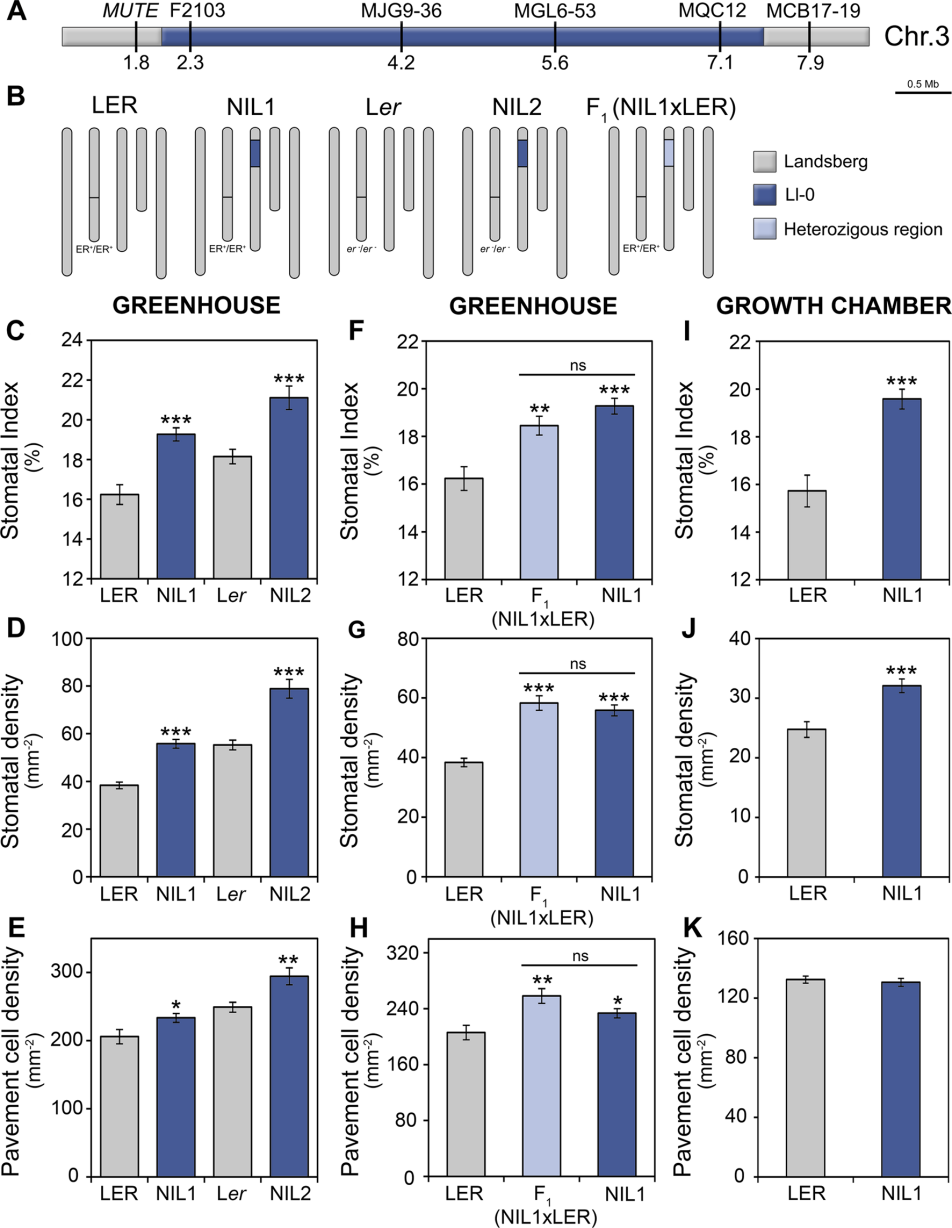
Characterization of stomatal abundance related phenotypes of *MID3* in NILs. **(A)** Physical map of the *MID3* region on chromosome 3, indicating marker names and positions in megabases (Mb). **(B)** Graphical genotypes of NILs, their reference strains and the F_1_ hybrids. NILs carried Ll-0 alleles at the *MID3* region in L*ER* (*ER*; NIL1) or L*er* (*er-1*; NIL2) background. The color code indicates homozygous and heterozygous regions for Landsberg and Ll-0 alleles. **(C**–**K)** Stomatal abundance related traits in the adaxial cotyledon epidermis of 21 dag plants. Stomatal index **(C**, **F**, **I)**, stomatal density **(D**, **G**, **J)**, and pavement cell density **(E**, **H**, **K)** were scored in the genotypes and growth environments indicated. Each panel shows the mean ± SE of 10–20 plants. Significant differences (***P ≤ 0.001; **P ≤ 0.01; *P ≤ 0.05; Student's *t*-test) with respect to the reference strain (L*ER* or L*er*) are indicated by asterisks. ns, not significant.

In order to confirm the effects of the *MID3* genomic region on epidermal cell traits, we developed two NILs carrying a single Ll-0 introgression fragment in the Landsberg genetic background (**Figures 3** and **5A, B**), but differing in the *ER* allele (**Figure 5B**). Analysis of these lines showed that NIL1 and NIL2 have significantly higher values for the three epidermal traits than their corresponding reference genotypes L*ER* and L*er*, in agreement with the *MID3*-Ll-0 allele effects estimated in the QTL mapping (**Figures 5C–E**). This result validates *MID3* effects on adaxial cotyledon traits and narrows down its location to a genomic region of ∼6 Mb, between positions 1.8 and 7.9 of chromosome 3 (**Figure 5A**). It is worth noting, however, that the estimates of *MID3* effects in both NILs were substantially higher for SI and lower for the cell-density traits than in the RIL population (see **Figures 5C–E** and **Table S4**). Moreover, comparison of the phenotypic effects in both NILs showed that *MID3* and *ER*/*MID2* acted additively for the tree traits (**Figures 5C–E**).

To characterize *MID3* we also analyzed the F_1_ (NIL1xL*ER*) progeny. These F_1_ plants showed similar phenotypes than NIL1 for all traits (**Figures 5F–H**) indicating that Ll-0 alleles at *MID3* are dominant over L*ER* alleles. Furthermore, to determine if *MID3* interacts with environmental factors, NIL1 was also assessed in growth chambers. In these conditions, the Ll-0 allele increased SI and SD (*P* < 0.001), as observed in greenhouse experiments, but it did not affect PD (**Figures 5I–K**). Therefore, *MID3* showed significant genotype-by-environment interactions for PD (**Table S5**) indicating that *MID3* cell-size effects are sensitive to environmental inputs.

### Fine Mapping of *MID3*

**Figure 6:**
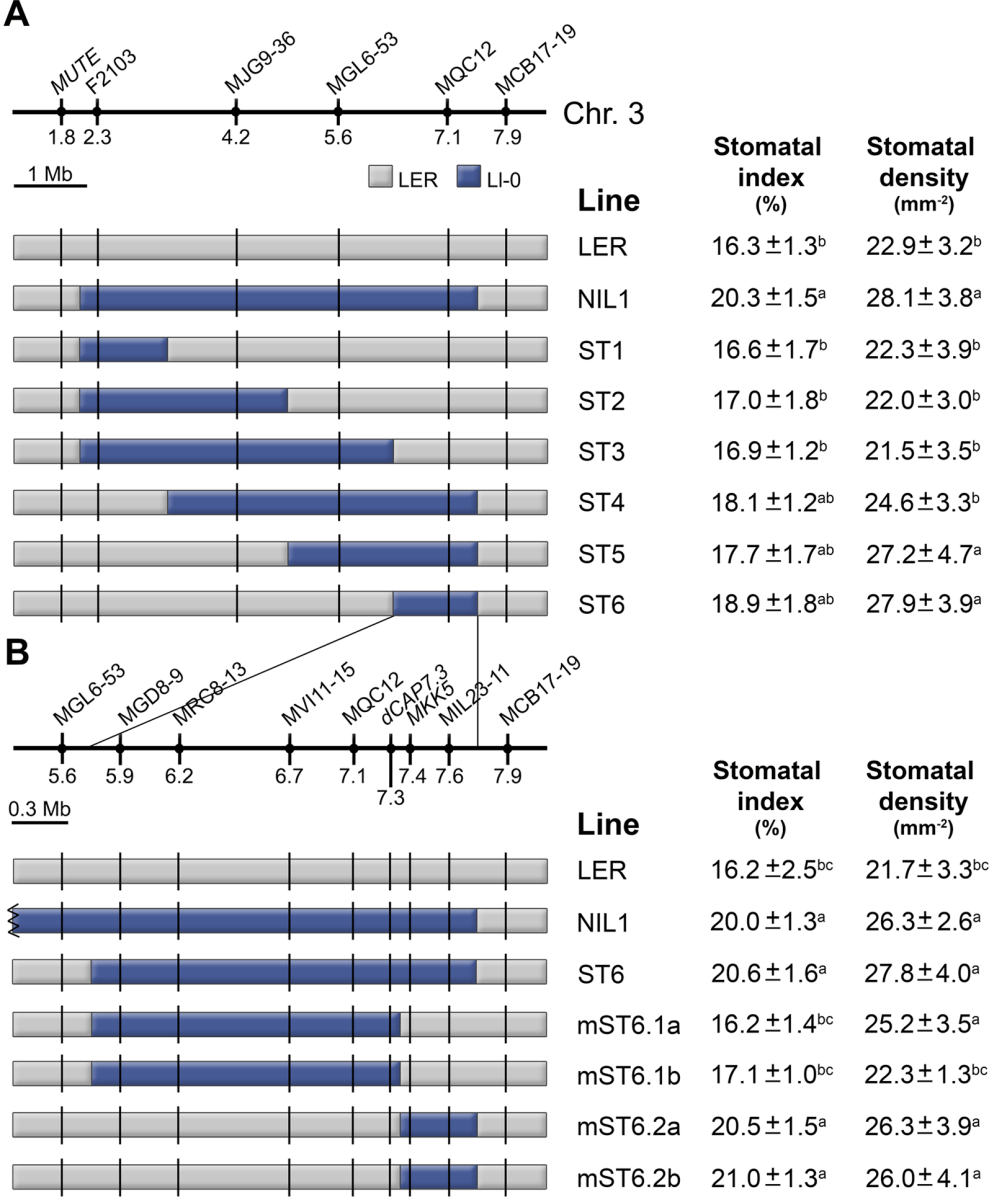
Fine mapping of *MID3*. **(A)** L*ER* lines with Ll-0 overlapping introgression fragments derived from NIL1. **(B)** The region introgressed in ST6 is expanded to show the newly developed markers and the precise recombinant breakpoints for proximal and distal mini-STAIR (mST) lines. Names and position (Mb) of markers are shown above each panel. Color codes indicate Landsberg (gray) and Ll-0 (blue) alleles. Stomatal index and density were scored in the adaxial cotyledon epidermis at 21 dag for all lines in growth chamber experiments. Each panel shows the mean ± SE of 10–15 plants. Significant phenotypic differences (*P* ≤ 0.05; Tukey's test) are indicated with letters. a: differing from L*ER*; b: differing from NIL1; c: differing from ST6.

To fine map *MID3* we developed a mapping population based on a STAIRS design (Stepped Aligned Inbred Recombinant Strains; Koumproglou et al., 2002). This STAIRS (ST) population consisted of six homozygous lines that carry partially overlapping Ll-0 fragments of the *MID3* region from NIL1, in a L*ER* background (**Figure 6A**). All ST lines with Ll-0 alleles at marker MQC12 (ST4, ST5 and ST6) did not differ significantly from NIL1 in any trait examined, while lines with L*ER* alleles at MQC12 (ST1, ST2, and ST3) were phenotypically similar to L*ER*. These results located *MID3* in a genomic region of ∼2.5 Mb, between positions 6.35 and 7.5 Mb (**Figure 6A**).

To find candidate genes for *MID3* we analyzed this genomic region searching for genes previously involved in stomata development. Interestingly, *MUTE* and *FAMA* (Pillitteri et al., 2007; Ohashi-Ito and Bergmann, 2006) were excluded because they are located in the chromosome three distal region outside the *MID3* location. However, this region encompasses the *MKK5* gene, which is known to negatively regulate stomatal numbers (Wang et al., 2007). Then, we used ST6 to develop a new set of NILs carrying recombination events around the *MKK5* locus (**Figure 6B**). Homozygous lines carrying proximal or distal regions of the ST6 introgression were named as mini-STAIRS (mST). Phenotypic analyses showed that lines mST6.2a and mST6.2b carrying Ll-0 proximal introgressions that span *MKK5* displayed high SI and SD values similar to ST6 and NIL1 (**Figure 6B**). Accordingly, lines mST6.1a and mST6.1b bearing the distal Ll-0 segment, i.e., *MKK5*-L*ER* allele, displayed similar SI and SD to L*ER*. Therefore, the *MID3* gene(s) was mapped to a ∼600 kb genomic region overlapping with *MKK5*. Sequencing of the *MKK5* coding region of Ll-0 and L*er* identified two nucleotide polymorphisms, none of them resulting in amino acid substitutions (**Table S6**). Hence, if *MKK5* underlies *MID3*, functional differences between Ll-0 and L*er* alleles may arise from cis-acting regulatory polymorphisms but not from changes in protein structure.

We finally tested if the small mapping region of *MID3* also affects stomata abundance traits in greenhouse conditions. In this environment, NIL1, ST6, and mST6.2a had similar SI, SD, and PD but significantly higher than those of L*ER* (**Figure S4**), supporting the pleiotropic effects of *MID3* on cell-type proportion and density.

### Stomata Developmental Processes Regulated by *ERECTA* and *MID3*

**Figure 7:**
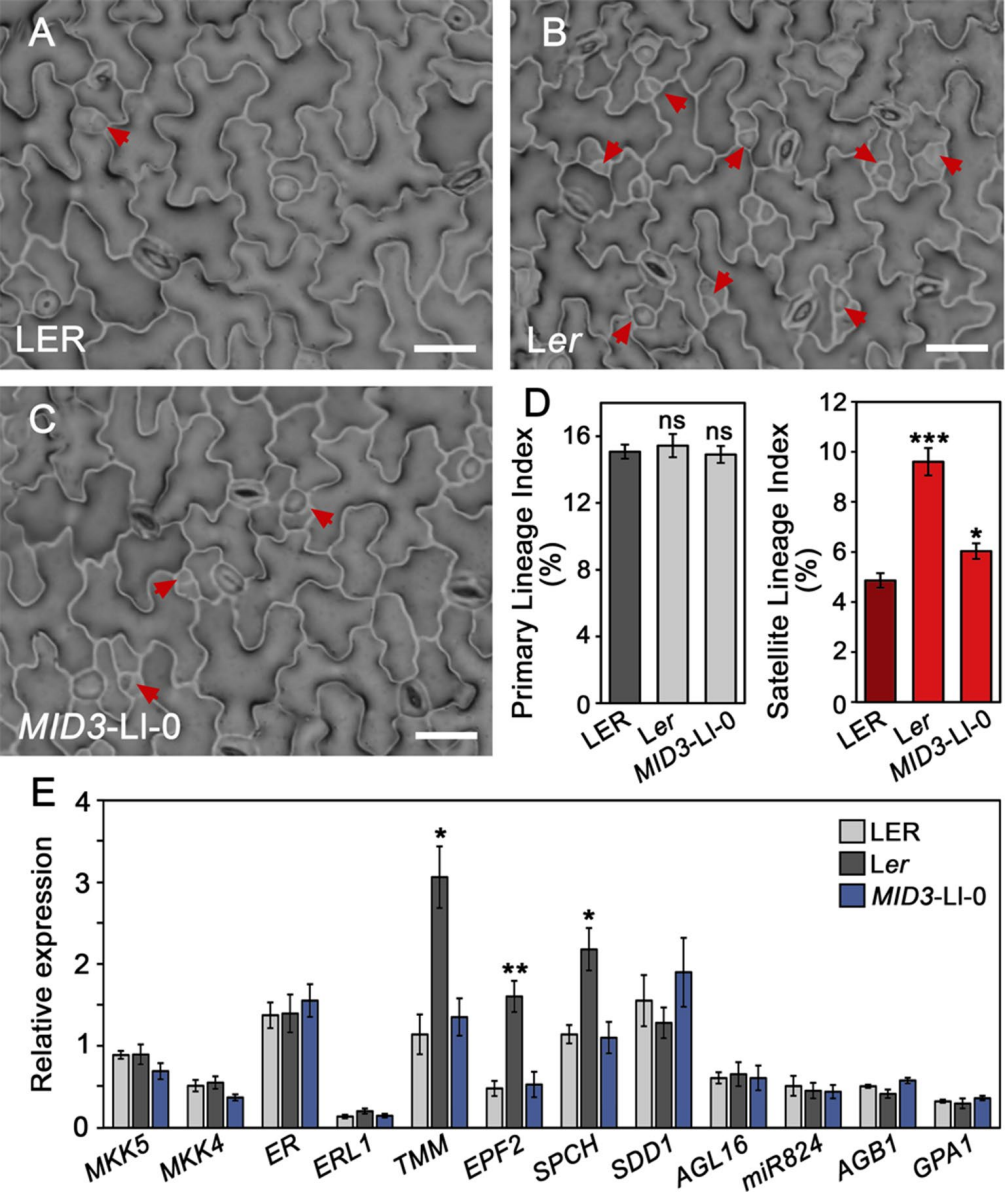
Differential regulation of stomatal processes and gene expression by *MID3* and *ER*. **(A**–**D)** Indexes of primary and satellite stomatal lineages were scored in the adaxial cotyledon epidermis of 3 dag seedlings for the indicated genotypes. **(A**–**C)** show representative epidermis from the plants scored; red arrows indicate satellite lineages. **(D)** Mean ± SD of primary (gray) and satellite (red) lineage indexes obtained from 10 to 15 plants. Asterisks indicated significant differences (***P ≤ 0.001; *P ≤ 0.05; Student's *t*-test) with respect to L*ER*. ns, not significant differences. **(E)** Relative gene expression determined by qPCR for the indicated genes in 3 dag seedlings of L*ER*, L*er*, and *MID3*-Ll-0 lines. Bars show the mean ± SE of three biological replicates. Asterisks indicated significant differences (**P ≤ 0.01; *P ≤ 0.05; Student's *t*-test) with respect to L*ER*.

Plants carrying the *MID3*-Ll-0 or the *er-1* alleles display a SI increase in the mature adaxial cotyledon epidermis as compared to L*ER*. These phenotypes could arise from changes in the occurrence of primary stomatal lineages initiated from the protodermis, or from their increased satellization at later stages of epidermal development. The two processes are under distinct genetic control as they present independent variation in natural *Arabidopsis* accessions (Delgado et al., 2011), and some genes have been reported to specifically control satellization in *Arabidopsis*. To examine whether *ER* and *MID3* differentially regulate these initiation events we measured the relative contribution of primary lineages (PL) and satellite lineages (SL) to the total lineages (TL) formed at 3 dag in adaxial cotyledons of L*er* (*er-1*) and the *MID3-*Ll-0 mini-ST line mST6.2b with respect to L*ER* (**Figures 7A–D** and **Table S7**). At this developmental time, SL were already abundant and easily identified in all genotypes (see material and methods for identification criteria). While *er-1* or *MID3*-Ll-0 alleles did not significantly affect the PL index (PLI), they both led to an increase of the SL index (SLI), particularly strong in the *er-1* (L*er*) mutant. Accordingly, SL contribution to total stomatal lineages (%SL) raised by about 30% in L*er* and 10% in *MID3*-Ll-0 line with respect to L*ER* (**Table S7**). Reiterated satellization was undetectable in L*ER* and *MID3*-Ll-0 line, while L*er* showed a small but significant proportion of reiterated satellization (above 4%). Despite L*er* and the *MID3*-Ll-0 line had similarly higher SI than L*ER* in mature organs, L*er* showed a TL index (TLI) much higher than the *MID3*-Ll-0 line and L*ER* at 3 dag, while *MID3*-Ll-0 differed only marginally from L*ER*. Hence, we compared PL and SL abundance in *MID3*-Ll-0 and L*ER* at a later developmental time (5 dag) (**Table S8**). In L*er*, the over-proliferating epidermal cells characteristic of the *er-1* mutation prevented a clear SL quantification at this stage. At 5 dag, TLI was already larger in the *MID3*-Ll-0 line than L*ER*, consistent with their SI at maturity, and the overproduction of SL by *MID3*-Ll-0 was the only process contributing significantly to such increase. Taken together, these data indicate that *ER* and *MID3* control the spacing divisions that initiate SL in opposite directions: the functional *ER* allele limits these divisions, while the dominant *MID3*-Ll-0 allele promotes them.

### Effect of *MID3 *and *ERECTA* Alleles on the Expression Profile of *S*tomatal *G*enes

To test whether *MID3* and *ER* might control spacing divisions by regulating the expression of distinct key stomatal regulators, we measured gene expression levels by qPCR in 3 dag seedlings of the *MID3-*Ll-0 line mST6.2b, L*er*, and L*ER* (**Figure 7E**). At this stage, most developing stomatal lineages in the seedling come from cotyledons and, as shown above, the three genotypes already differ in the relative proportions of PL and SL (**Figures 7A–D** and **Table S7**). The set of genes analyzed included positive (*SPCH*) and negative (*ERL1*, *EPF2, TMM*, and *SDD1*) general regulators of stomatal lineage initiation and/or progression, as well as specific regulators for SL formation. The latter include the satellization-promoting factor *AGL16* and its down-regulator miR824, a pathway that enhances satellization, and also *AGB1* and *GPA*, known to reduce the occurrence of satellite stomata (Kutter et al., 2007; Zhang et al., 2008; Yang et al., 2014). In addition, we evaluated the expression of the candidate gene for *MID3*, *MKK5*, and its functionally redundant paralog *MKK4*, both *MKK* genes acting as general negative regulators of stomatal numbers (Wang et al., 2007; Lampard et al., 2009). Transcript levels for the satellization factors (*AGL16*, *miR824*, *AGB1*, and *GPA1*) did not differ among the *MID3*-Ll-0 line, L*er*, and L*ER*. A weak decrease in *MKK5* and *MKK4* transcript levels was detected in the *MID3-*Ll-0 line compared to L*ER*, though the changes were no statistical significant (*P* = 0.14). Therefore, these results do not clarify if *MKK5* is the gene underlying *MID3*. The remaining general regulators of stomata development did not differ significantly between *MID3-*Ll-0 line and L*ER*. However, expression levels of *SPCH*, *EPF2*, and particularly *TMM*, were much higher in L*er* than L*ER* or the *MID3-*Ll-0 line. Intriguingly, *ER* did not affect its own expression level or the SPCH-target genes *ERL1* and *SDD1*, whose transcript levels have been previously correlated with the abundance of developing stomatal lineages in most genotypes and environments (Lau et al., 2014)

## Discussion

### Genetic Basis of the L*er/*Ll-0 Variation for *S*tomatal Traits

Based on the analysis of cell-type proportion and density traits in the adaxial epidermis of cotyledons and first leaves, we have previously documented a wide natural genetic variation for stomatal abundance in *A. thaliana* (Delgado et al., 2011). Here, we dissected the genetic bases for the distinct stomatal phenotypes of Ll-0 and L*er*, focusing on their adaxial cotyledon epidermis. The analysis of reciprocal F_1_ hybrids indicates different contributions from the zygotic and maternal genotypes on the various traits. While SI was controlled by the zygotic genotype, PD was under a strong maternal influence. Both effects combined for SD, as expected from the equal contribution to SD values of stomatal development and cell size (measured by SI and PD, respectively). Whether these maternal genetic effects persist in true leaves or they relate to the known maternal control of early seedling growth (Lemontey et al., 2000) remains to be determined.

Phenotypic variation in the Ler×Ll‐0 RIL population exhibited a robust genetic basis for all traits suggestive of a multigenic control, with opposite allelic effects from both parental accessions. Broad-sense heritability for SI was particularly high when compared with natural accessions (66.6 *vs.* 33–49%, respectively; see Delgado et al., 2011), suggesting that genes controlling stomatal developmental processes, and not just epidermal cell sizes (as measured by PD), underlie the stomatal differences between Ll-0 and L*er*. Notably, some RILs displayed transgressive phenotypes with very high values for all traits, which, however, did not concur with aberrant stomatal patterns. This suggests that the genetic variants segregating in the RILs control stomatal production without interfering with cell-to-cell signaling mechanisms involved in proper stomatal spacing (reviewed by Zoulias et al., 2018; Lee and Bergmann, 2019).

Our QTL mapping in this RIL population identified five genomic regions affecting stomatal abundance. The three *MID* regions contain the identified QTL for SI, all them overlapping with QTL for SD and PD. Such phenotypic effects fit with those expected for loci modulating stomatal development and having an additional effect on pavement cell size. In the two *MDC* regions, only QTL for SD and PD co-located, suggesting that *MDCs* influence SD by regulating the size of pavement cells. Interestingly, such distinct *MID* and *MDC* effects agree with the effects described for genes controlling both initiation and cell-divisions in the stomatal lineages, the expansion of pavement cells, or both processes (Asl et al., 2011; Sablowski 2016; Fox et al., 2018). *MID* and *MDC* regions showed mostly small additive effects on the various traits, with the exception of *MID2*, which explained more than 50% of the phenotypic variance of all traits. Consistent with the correlations among traits, *MID* regions affect SI, SD, and PD values in the same direction, as *MDC* regions also do for SD and PD. Therefore, these allelic variants seem to allow for compensatory mechanisms that counterbalance cell size and cell numbers in the leaf epidermis, integrating the division activity of stomatal lineages (Geisler and Sack, 2002; Tsukaya, 2008). Taken together, these results indicate that Ll-0 and L*er* differences in stomatal abundance traits are due to a small number of loci that likely regulate stomatal development processes and/or cell size by partly independent pathways.

Since natural variation in stomatal development has barely been studied, we focused in the characterization *MID2* and *MID3* by genetic, developmental, and molecular approaches. Our results confirmed that *ER* is the gene underlying *MID2,* as determined by the *er-1* loss-of-function allele present in L*er* parental accession (Torii et al., 1996). We also validated *MID3* effects and mapped the underlying gene(s) to a ∼600 kb region. As discussed in the next sections, the *MID2*-L*er* (*er-1*) and *MID3*-Ll-0 alleles lead to large increases in SI, SD, and PD that resulted from combining higher stomatal lineage initiation with reduction of pavement cell sizes. These findings demonstrate the value of our strategy to identify loci directly involved in stomata developmental processes. Moreover, *MID3*-Ll-0 provides the first description of a natural allele with a large impact on stomatal numbers demonstrated to regulate stomatal development. In fact, when isolated as a single introgression in a L*ER* background, *MID3*-Ll-0 SI and SD values were nearly identical those produced by the *MID2*-L*er* allele. Most likely, the under-estimation of *MID3* effects in the RIL population is due to the segregation of multiple QTL in the RIL population, which increases the residual variance at each QTL under study (Keurentjes et al., 2007). Furthermore, both *MID2* and *MID3* loci differ in their genetic and environmental interactions. *MID3*-Ll-0 allele behaves as dominant, and shows significant genotype-by-environment interactions for PD. By contrast, *MID2/ER*, is semidominant for SI and not affected by the environmental factors in our conditions. Finally, *MID3* and *MID2*/*ER* showed additive effects on the three traits. Therefore, *MID3* and *MID2*/*ER* seem to control stomatal development and numbers by different regulatory pathways.

### *ERECTA* Shows Differential Effects on Cell-Type Proportions and Densities of Both Cotyledon Epidermes

*ER* encodes a leucine-rich repeat receptor-like protein kinase with a complex functional redundancy in stomatal development with its paralogs *ERL1* and *ERL2* (Shpak et al., 2005). However, only *ER* displays a major role in restricting the entry divisions that initiate stomatal cell lineages (Shpak et al., 2005). Upon EPF2 peptide binding, ER activates the YDA-leaded MAPK cascade, which down-regulates SPCH activity inhibiting the entry divisions (Lampard et al., 2008; Lee et al., 2012; Lee et al., 2015). *ER* also promotes the growth of epidermal cells by increasing their ploidy levels through E2Fa regulation (Guo et al., 2019).

The phenotypic characterization carried out in this study shows that all three *er* loss-of-function mutant alleles have increased stomatal and pavement cell densities in both, adaxial and abaxial, epidermis of cotyledons. However, while the SI also increased in the adaxial side, it decreased in the abaxial epidermis. The lack of *ER* function has been previously reported to produce two developmental effects on the abaxial cotyledon, which impinge on SI in opposite ways. It increases stomatal initiation (which would rise both SLGCs and stomata numbers) but, since a substantial proportion of the lineages failed to differentiate into stomata, the net balance is a reduction of the number of stomata with respect to the SLGCs produced, and a decreased SI (Shpak et al., 2005). Our abaxial data confirm the presence of arrested linages and a decreased SI in all the *er* mutants studied. In the adaxial cotyledon epidermis, however, we did not observe arrested linages and, since stomatal initiation increases (see next section), SI increases. We cannot rule out that changes in amplifying divisions, which decrease SI but increase the pool of SLGCs amenable to undergo spacing divisions, contribute to the observed *er* phenotypes. In addition, the satellite lineages might undergo fewer amplifying divisions, perhaps because they take place at later developmental times, when the division competence of the lineages is limited (Geisler and Sack, 2002). Furthermore, our results show that ER is only necessary for lineage progression in the abaxial epidermis and suggest differences in developmental signals between the adaxial and abaxial domain. Interestingly, mutations in *TMM*, a stomatal receptor that works in complexes with *ER*, also lead to more severe phenotypes in the abaxial than the adaxial side (Geisler et al., 1998). Nevertheless, the opposite SI phenotypes found here for *er* cotyledons contrast with other reports in late rosette leaves, where no change in adaxial SI or an increase in abaxial SI was observed (Masle et al., 2005; Tisné et al., 2011; Jordá et al., 2016). Differences in the developmental contexts of cotyledons and leaves and/or in growth conditions may account for these disparities.

Despite de involvement of ER in many developmental and physiological processes that impact plant performance and survival, current knowledge on natural allelic variation in the *ER* locus is surprisingly limited. In fact, dysfunctional *ER* alleles have been reported only in the Van-0 and Hir (Hiroshima) accessions (van Zanten et al., 2009), and their effects have been studied for just a few processes. Our results show that *ER* loss-of-function contributes to the high stomatal values of Van-0 adaxial cotyledons. *ER* dosage affects SI but not PD values, indicating that stomatal developmental processes are sensitive to ER levels. This result agrees with the *ER* role in fine-modulating the accumulation of SPCH (Lee et al., 2012; Lee et al., 2015), which in turn drives stomatal production. However, it remains to be determined if effects of natural *ER* alleles on stomatal traits provide any adaptive advantage under natural environments, as suggested in wild beans, where some SNP haplotypes at an *ER* homologue appeared associated with drought tolerance (Blair et al., 2016).

### *MID3* and *ERECTA* Modulate Satellite Stomatal Lineages

We have shown that *ER* and *MID3* influence stomatal production by modulating the incidence of satellite stomata, which implies that both loci regulate the competence of SLGCs to execute spacing divisions. *MID3* effect appears to be restricted to first order SL, while *ER* showed an additional role in SL re-iteration. However, our data do not exclude that *ER* and *MID3* may have an additional function on PL initiation, which can occur at later times and could be especially relevant for *er-1*, as this allele expands the time-window of cell division competence in the leaf epidermis (Tisné et al., 2011). Notably, the *MID3*-Ll-0 allele promotes spacing divisions in a dominant manner, in contrast with the *ER* repressive role. Multiple studies have addressed how *ER* negatively regulates SPCH abundance to restrict entry divisions (reviewed in Endo and Torii, 2019), but little is known about its involvement in other processes during stomatal developmental. Lineage arrest in *er* mutants supports the idea that *ER* is expressed in SLGCs to buffer the inhibitory activity of EPF1 in the neighboring meristemoid (Qi et al., 2017). Our results show that *er-1* increased spacing divisions, providing a novel *ER* role in limiting the stomatal fate of SLGCs. This new role of *ER* fits well with the negative program that differentially inhibits both SPCH expression and division potential in SLGCs, needed to assign distinct cell fates after lineage asymmetric divisions (Robinson et al., 2011). Recent studies have addressed the mechanisms involved in this SLGC repression and how it could be counterbalanced to allow spacing divisions (Zhang et al., 2015; Zhang et al., 2016; Houbaert et al., 2018; Vatén et al., 2018). These reports evidenced a main role of the YDA-MAPK module in SLGC fate decision, associating high levels of MAPK activity with pavement cell differentiation, while low levels associated with spacing divisions. Interestingly, *MID3* fine mapping identified the *MKK5* gene, which encodes a component of this MAPK module, as a candidate for *MID3*. Sequencing of the *MKK5* coding region ruled out functional differences between the MKK5 protein in Ll-0 and L*er*. We then tested if *MKK5 cis*-regulatory polymorphisms could underlie *MID3* locus, but we could not detect significant expression differences between *MID3*-Ll-0 line and L*ER*. While these results do not excluded that *MKK5* underlies the *MID3*-Ll-0 phenotype, they do not provide support for this possibility either. Therefore, the possible causal relationship between *MID3* and *MKK5* remains to be determined. Nevertheless, the effect of the natural *MID3*-Ll-0 allele illustrates the large impact that a single genetic factor can have on stomatal numbers by promoting SL. Indeed, *MID3*-Ll-0 increased SI by a 17%, and SD by about a 45%, while maintaining a proper stomatal pattern, with no observable pleiotropic effect on plant growth and reproduction.

Our genetic, developmental and expression analyses suggest that *MID3* and *ER* act through different regulatory pathways to control satellite stomata frequency. First, they exhibited additive effects for SI, a trait that reflects the frequency of SL. Second, *MID3* and *ER* differ in the timing of their developmental action because *er-1* effects on satellite and total SI appears earlier during cotyledon development than *MID3*-Ll-0 effects. Third, they also show differential gene expression patterns since *er-1* showed increased expression of general regulators of stomatal lineage progression (*SPCH*, *EPF2*, and *TMM*), while *MID3*-Ll-0 had no detectable change at 3 dag. Expression changes in these genes could simply represent the epidermal phenotype of L*er* and *MID3*-L1-0, as increased number of the cell types (stomatal precursor cells) which express *SPCH*, *EPF2*, and *TMM* would increase the level of these transcripts. Interestingly, neither *MID3* nor *ER* altered the expression of specific regulators of SL production (*AGL16*, *miR824*, *AGB1*, and *GPA1*; Kutter et al., 2007; Zhang et al., 2008). Thus, *MID3* and *ER* might operate through pathways that do not involve such regulators. Recently, cytokinins have been shown to promote spacing divisions in SLGCs through re-activation of SPCH expression, which increases total stomata by 10% (Vatén et al., 2018). Therefore, *MID3* and *ER* might also impinge on SL initiation through still undescribed pathways. It has been assumed that PL and SL contribute to the phenotypic plasticity of stomatal development in response to internal and environmental signals (Bergmann and Sack, 2007; Casson and Hetherington, 2010; Lampard, 2010). Supporting this view, recent work on the stomatal development responses (Haus et al., 2018) highlights the importance of SL in developmental plasticity in response to the atmospheric CO_2_ concentration.

Although satellization can contribute to as much as 35% of adaxial and 65% of abaxial stomata in Col-0 cotyledons, (Geisler and Sack, 2002), few studies have addressed this process known to result from highly specific asymmetric divisions that are different from the entry or amplification divisions. Our work demonstrates a direct role of *ER* in SL initiation. In Delgado et al. (2011)we reported that satellization underlies a relevant fraction of the natural variation observed in stomatal abundance among wild accessions. In fact, some accessions showing similar SI values differed in satellite index. In that work, we substantiated the existence of natural variation for both PL and SL initiation, and suggested that genetically independent pathways govern the two processes. Here, we confirmed the existence of natural variation for SL initiation, and identified a locus, *MID3*, directly involved in SL initiation through pathways partially different from *ER*. Thus, our findings on *MID3* indicate that genetic modulation of satellization is a component of the variation for stomatal abundance among natural populations, which might contribute to plant adaptation.

## Data Availability Statement

All datasets for this study are included in the article/**Supplementary Material**.

## Author Contributions

DD, CA-B, and MM designed the experiment with contributions of CF. CA-B and MM supervised the work. DD performed most of the experimental work with contributions from ES-B in RIL experiments and QTL analysis, AM in qPCRs assays, and CM-J in obtaining *MKK*5 sequences. All authors analyzed the data. MM and CA-B wrote the manuscript with the contribution of CF and DD.

## Funding

MM's and CF's lab has been funded by grants AGL2015-65053-R from the Spanish Government and PPII10-0194-4164 and SBPLY/17/180501/000394 from Junta de Comunidades de Castilla-la Mancha and by UCLM intramural grants. EU FEDER funds complemented all these grants. CA-B's lab has been funded by grant BIO2016-75754-P from the Agencia Estatal de Investigación (AEI) of Spain and the Fondo Europeo de Desarrollo Regional (FEDER, UE).

## Conflict of Interest

The authors declare that the research was conducted in the absence of any commercial or financial relationships that could be construed as a potential conflict of interest.
