# Supplementary material for: A Genetic Dissection of Natural Variation for Stomatal Abundance Traits in Arabidopsis
Source: Front Plant Sci. 2019 Nov 11;10:1392. doi: 10.3389/fpls.2019.01392 (PMC6859887; doi:10.3389/fpls.2019.01392)
Supplement: Supplementary file 1 [file Image_1.pdf]

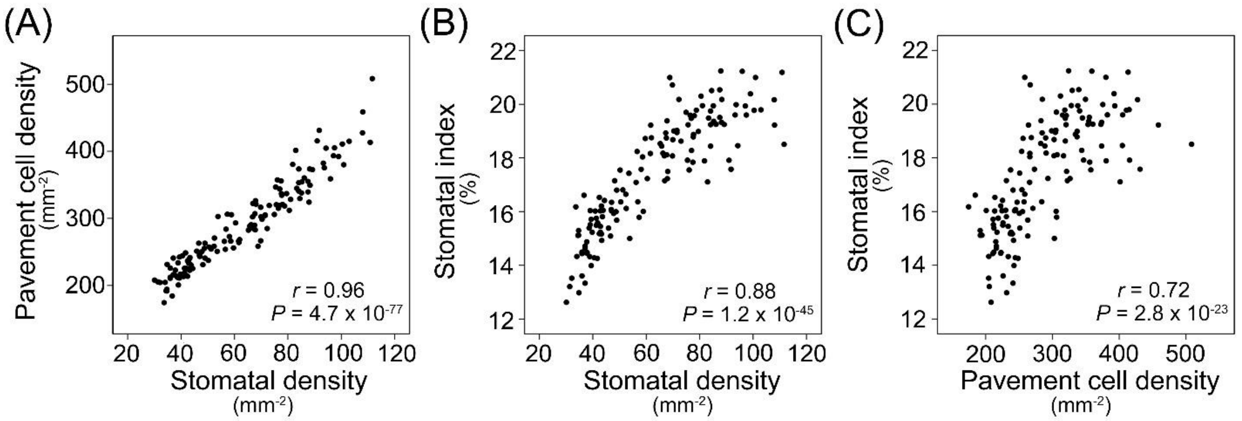

Supplementary Figure S1. Trait correlation in the L*er x* Ll-0 RIL population. (A–C) Scatter plots showing the relationship between mean trait values of the 139 RILs. Pearson's coefficient (*r*) and *P* values are indicated.
