# Supplementary material for: A Genetic Dissection of Natural Variation for Stomatal Abundance Traits in Arabidopsis
Source: Front Plant Sci. 2019 Nov 11;10:1392. doi: 10.3389/fpls.2019.01392 (PMC6859887; doi:10.3389/fpls.2019.01392)
Supplement: Supplementary file 2 [file Image_2.pdf]

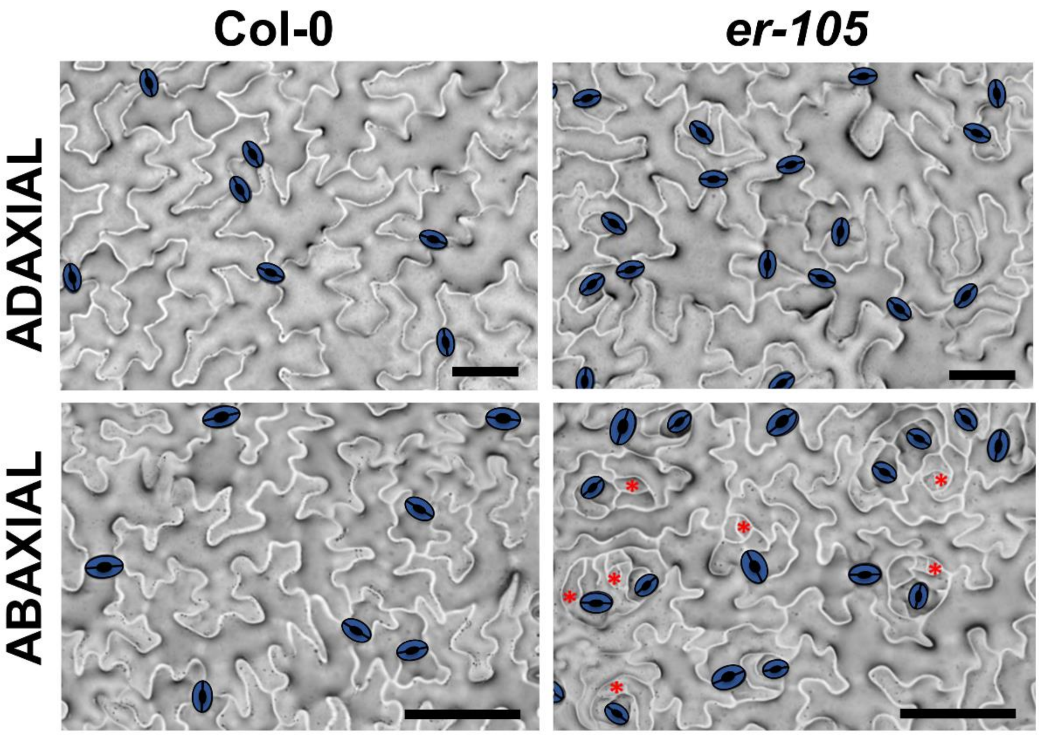

Supplementary Figure S2. *er-105* differential phenotypes in both cotyledon epidermis. Representative adaxial and abaxial epidermis of Col-0 and *er-105* mature cotyledons, with stomata false-coloured in blue. Note the presence of arrested lineages (red asterisks) only in the abaxial epidermis of *er-105*. Micrographs were obtained with the dental resin method. Scale bars are 100 µm.
