# Supplementary material for: A Genetic Dissection of Natural Variation for Stomatal Abundance Traits in Arabidopsis
Source: Front Plant Sci. 2019 Nov 11;10:1392. doi: 10.3389/fpls.2019.01392 (PMC6859887; doi:10.3389/fpls.2019.01392)
Supplement: Supplementary file 3 [file Image_3.pdf]

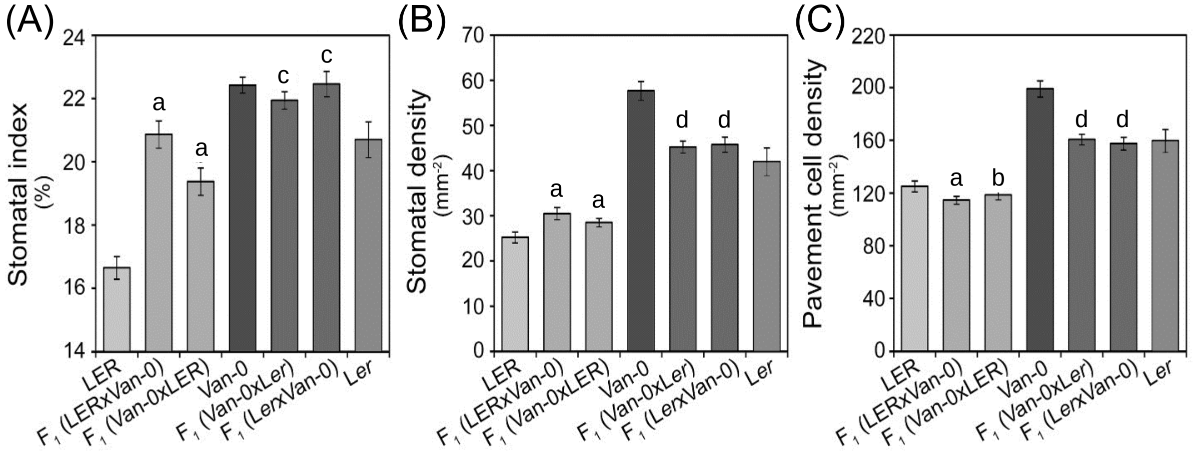

Supplementary Figure S3. Effect of the *erecta* allele of Van-0 on stomatal abundance traits. Stomatal index and density and pavement cell density were scored in adaxial cotyledon epidermis of Van-0, L*er*. L*ER* and the indicated F_1 hybrids. For each trait, the mean ± SE of 10 plants are presented. Letters over the bars refer to comparisons of a F_1 hybrid with its parents by a Student's *t*-test, according to the following code; a: differing from L*ER* and Van-0; b: differing from Van-0 but not from L*ER*; c: differing from L*er* but not from Van-0; d: differing from Van-0 but not from L*er*.
