# Supplementary material for: A Genetic Dissection of Natural Variation for Stomatal Abundance Traits in Arabidopsis
Source: Front Plant Sci. 2019 Nov 11;10:1392. doi: 10.3389/fpls.2019.01392 (PMC6859887; doi:10.3389/fpls.2019.01392)
Supplement: Supplementary file 4 [file Image_4.pdf]

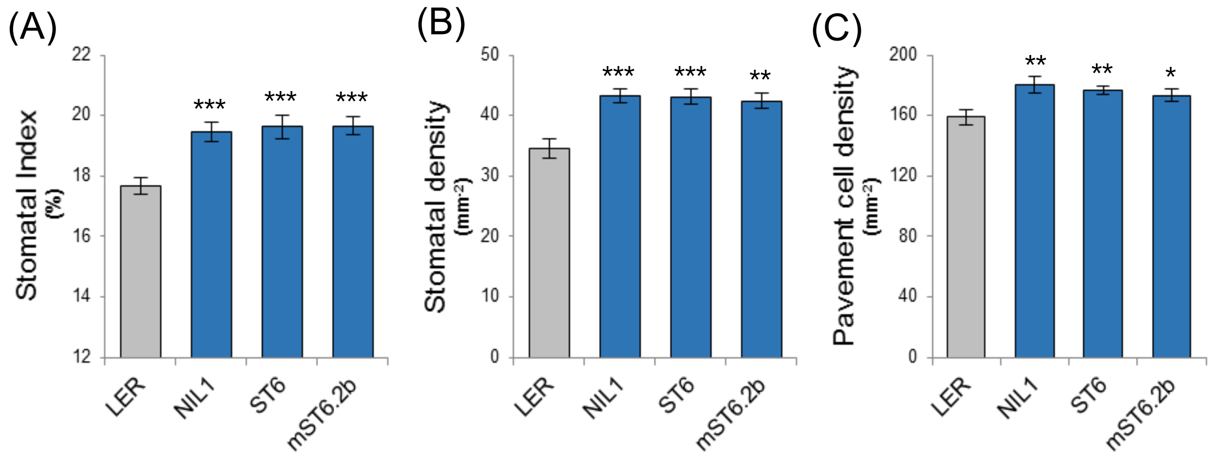

Supplementary Figure S4. Trait values of *MID3*-Ll-0 lines grown in greenhouse. Stomatal index (A), stomatal density (B) and pavement cell density (C) were scored at 21 dag in the adaxial cotyledons of NIL1, ST6, mST6.2b and L*ER* plants growth in greenhouse. Each panel shows the mean±SE of 10 plants. Significant differences with respect to the reference strain L*ER* are indicated by asterisks (****P* ≤ 0.001; ***P* ≤ 0.01; * P ≤ 0.05; Student's *t*-test). Note that in greenhouse conditions the *MID3*-Ll-0 allele increased SI, SD and also PD.
